# Developing a model of interactive health literacy among college students majoring in kinesiology: a grounded theory approach

**DOI:** 10.3389/fpubh.2025.1510035

**Published:** 2025-04-30

**Authors:** Miaomiao Wen, Xiaorui Wang, Mei Zhou, Zhihua Yin, Shuaiqiang Yuan, Shaoqiang Liu

**Affiliations:** ^1^College of Physical Education and Health, East China Normal University, Shanghai, China; ^2^Department of Social Sports, Hebei Sport University, Shijiazhuang, China; ^3^Department of General Education, Shanghai Urban Construction Vocational College, Shanghai, China; ^4^School of Physical Education, Hebei Normal University, Shijiazhuang, China

**Keywords:** kinesiology, interactive health literacy, grounded theory, sports, China

## Abstract

**Background:**

Health literacy among college students majoring in kinesiology is higher than that among students in other majors. However, the structure of interactive health literacy is poorly understood, which may affect overall health literacy. Despite existing research on health literacy, studies targeting college students majoring in kinesiology are scarce, and a gap remains in translating study results into practical strategies. Therefore, this study constructed a theoretical model of interactive health literacy among college students majoring in kinesiology.

**Methods:**

Qualitative data were collected from online posts, a literature review, and semi-structured interviews with 11 college students majoring in kinesiology, physical education teachers, and six physical fitness experts in China. Participants were selected using purposive sampling. Interview questions focused on participants' perceptions of the structure and characteristics of interactive health literacy among college students majoring in kinesiology. Data were analyzed based on the grounded theory.

**Results:**

The analysis derived three categories with eight subcategories, 60 concepts, and 288 labels. The categories and subcategories were harnessing health information (comprising obtaining health information and comprehending health information), promoting health interactions (comprising perceiving health functions, shaping health awareness, and participating in health communication), and addressing health problems (comprising forming health motivation, making healthy decisions, and practicing healthy living).

**Conclusions:**

College students majoring in kinesiology in China exhibited unique trends in interactive health literacy compared to students in other countries. Further large-scale surveys are required to revise and validate the initial exploration of perceptions and investigate the structural system of interactive health literacy in various groups.

## 1 Introduction

Health literacy is an emerging area of research worldwide and can be used as both a goal for health education and health promotion and a method of assessing the outcomes of health education and health promotion efforts (1). Interactive health literacy focuses on the development of individual skills, such as problem solving, communication, making healthy decisions, and the ability to process health knowledge (2). Developing interactive health literacy requires health education activities in public places, such as schools and communities (3, 4). Kinesiology majors in this study refer to undergraduate studies in comprehensive, teacher training, and sports colleges and universities with physical education, athletic training, social sports instruction, and other related physical education and health disciplines as study majors. Participation in sports activities can promote students' health literacy (5), college students majoring in kinesiology usually have an understanding and awareness of health and strong health literacy. So the health literacy of college students majoring in kinesiology is higher than that of college students majoring in other disciplines. They are not only recipients but also future disseminators of health knowledge. In China, although the overall health education level of college students is high, their health literacy is generally low, indicating that they have not implemented health theories and skills or developed good living habits (6). Therefore, an interactive health literacy structural model for college students majoring in kinesiology would help colleges and universities conduct health education and promote students' physical and mental health.

Health literacy was first introduced as a policy issue in school health education in 1974, along with the observation that school health education was required to improve students' health literacy (5, 7). Health literacy has different definitions and conceptual models in public health and clinical medicine perspectives (8–10). However, both perspectives agree that health education plays an important role in clinical and community health literacy (11). Low health literacy is prevalent worldwide and affects individual health outcomes (12–15). People in some countries have little access to health education, are not equipped to prevent chronic disease (16), and are 1.5–3 times more likely to have poor health outcomes (17, 18). Health education in colleges can promote health literacy among students (19, 20) and enriches health behaviors and knowledge to equip them to cope with different health problems in their future lives (21, 22). Nutbeam categorized health literacy into three levels: functional, interactive, and critical (3). Interactive health literacy evaluates an individual's ability to access, understand, and apply health information in a specific context (23–25). To date, 32 health literacy scales have been developed, of which 21 can be used to assess interactive health literacy (26, 27). The Multidimensional Measure of Adolescent Health Literacy (MAHL) focuses on doctor-patient communication, interpersonal interactions, and the use of health information (28). Wallace et al. recommended a simple tool for screening patients with limited health literacy using one question (29).

China has emphasized the importance of health through the promulgation of a series of policies, such as the Outline of the “Healthy China 2030” Plan, the Opinions on Comprehensively Strengthening and Improving the Work of School Hygiene and Health Education in the New Era, and the Guidelines for Health Education in Ordinary Schools of Higher Learning. These policies indicate that improving health literacy is the most effective measure for fundamentally improving the health of the entire society. China's research on health literacy is in its infancy and focuses on health communication and the use of health education to improve the health literacy of the entire population (30). Comprehensive research on health literacy and its evaluation among various groups of people is lacking. College students majoring in kinesiology receive health education in college, and their daily life and behavior incorporate physical activities that improve health literacy. Based on the Healthy China strategy, college students must disseminate the concept of health and lead healthy lives. College health education and promotion aims to improve students' health literacy. The interactive health literacy of college students majoring in kinesiology is a key factor in improving the health literacy of the entire population.

Research on health literacy has examined various populations, such as adolescents, persons with disabilities, ethnic minority groups, and vulnerable groups, including migrant populations and left-behind children (31). Health education and promotion are internationally recognized as one of the main methods for improving populational health literacy, with improvements in health literacy used as a major indicator to reflect the effectiveness of health education and promotion (3). Improving the health literacy of college students majoring in subjects related to health and medicine is easier, and college students' health literacy is significantly influenced by their majors (32–34). As a special group with long-term exposure to sports, college students majoring in kinesiology engage in sports activities in their daily lives, thereby improving their health literacy. The health behaviors, habits, and knowledge that college students majoring in kinesiology obtain in college and their work after graduation are closely related to improving health literacy in the entire population. Examining the interactive health literacy of college students majoring in kinesiology would enrich the theoretical research on this topic and identify methods of improving college students' health literacy through health education. This would, in turn, promote college students' physical and mental health.

Although many studies have examined health literacy from various perspectives, and various programs have been proposed to improve it, studies targeting college students majoring in kinesiology are scarce. Moreover, most existing research focuses on the macro level of health literacy, and a gap remains in translating these results into practical strategies for interactive health literacy, limiting its comprehensive development. Previous studies have often used questionnaires; however, questionnaire surveys cannot be used to construct new theoretical models, as they use relatively simple questions to understand the current situation and existing problems. The grounded theory approach can be used to construct theoretical models from the bottom up based on the participants' experiences, opinions, and behaviors, providing a comprehensive understanding of their perceptions and understanding. Understanding is continually refined during the data collection process. The underlying logic of the grounded theory is to condense data and build theory from the bottom up. Researchers do not make prior assumptions but directly initiate data generalization and analysis. Therefore, ensuring the relevance and validity of the data during the data collection process is important.

In China, students majoring in kinesiology have limited knowledge of interactive health literacy. They use sports for personal health promotion and development without understanding their contribution to improving health literacy. A theoretical model could help college students majoring in kinesiology understand interactive health literacy and provide a scientific framework for their professional development. Therefore, this study used the grounded theory approach to construct a structural model of interactive health literacy among college students majoring in kinesiology. This model could help utilize and reflect the professional advantages of health promotion among college students majoring in kinesiology, advance research on evaluations of health literacy, and introduce a new perspective for health literacy research.

## 2 Materials and methods

This study has been approved by the Ethics Board at East China Normal University (HR 096-2021, 14 March 2021). Written informed consent was obtained from the individuals for the publication of any potentially identifiable images or data included in this article. Informed consent was obtained from all participants and the research team maintained the confidentiality of participant information involved. We certify that the study was performed in accordance with the 1964 declaration of HELSINKI and later amendments. All interviewees have already signed the interview opinion request form and agreed before the interview. The interview process will be recorded and their privacy will be ensured not to be leaked.

The textual data used for grounded theory in this study mainly comes from literature related to the research topic and textual data collected through interviews. First, a literature search was conducted using Chinese and international databases, such as China National Knowledge Infrastructure (CNKI) and Web of Science, to collect relevant information on interactive health literacy among college students majoring in kinesiology. We retrieved 39 and 195 relevant articles in Chinese and English, respectively, and analyzed the results to distill initial concepts and provide support for the theoretical framework. Second, semi-structured interviews were conducted with college students majoring in kinesiology and experts in the field of kinesiology and health. The interview formats included face-to-face offline interviews and social media (e.g., WeChat and Tencent Meeting) online interviews. After the interview, the interview process was converted into textual data. Finally, the collected relevant literature and interview text materials will be used to construct an interactive health literacy structure model for college kinesiology students using the grounded theory method.

### 2.1 Participants

In research based on the grounded theory, the appropriate sample size is determined based on data saturation (35, 36). Saturation is reached when no new relevant data emerge (37). Previous studies suggest that a sample size of 10–20 participants is sufficient in grounded theory research to generate a rich and detailed understanding of a phenomenon (38).

Participants were recruited through a combination of purposive and snowball sampling (39). Current research has shown that there are significant differences in the health literacy levels of people in different regions of China, mainly in the east, middle and west, gender and age, etc. (40). The core influencing factors that cause differences in the health literacy levels of people in different regions of China include social-economic factors, the environment and lifestyle, as well as the allocation of medical and health care resources (41, 42). Participants in this study came from all regions of China (East, North, South, West, Northeast, and Southwest), and in this way, covering the effects of different factors on the participants' health literacy levels, it can be inferred that the participants in the survey were reasonably representative of the target population. The inclusion criteria were adapted from Goodyear et al. (43), as follows: (1) studying kinesiology at a college; (2) being engaged in physical health education (defined as teaching the course); and (3) having knowledgeable about interactive health literacy. Finally, 11 students and six physical fitness specialists were included in the analysis. To ensure anonymity, each participant was assigned a numerical identifier from 1 to 17 (Tables 1, 2).

**Table 1:** Demographic characteristics of students (*N* = 11).

**No**.	**Gender**	**Class**	**College type**	**Specialization**	**Region**
1	Male	Freshman	Normal college	Sports training	Northeast
2	Male	Junior	Sports college	Sports training	Northeast
3	Female	Junior	Normal college	Physical education	Northwest
4	Female	Sophomore	Sports college	Sports training	Northeast
5	Male	Senior	Normal college	Sports training	Northwest
6	Male	Junior	Comprehensive college	The humanities of sport	Northwest
7	Female	Senior	Sports college	Physical education	Northwest
8	Female	Senior	Comprehensive college	Physical education	Northwest
9	Female	Freshman	Normal college	Kinesiology rehabilitation	Northwest
10	Male	Junior	Medical college	Kinesiology rehabilitation	Southwest
11	Male	Junior	Normal college	Sports ergonomics	Southeast

**Table 2:** Demographic characteristics of physical fitness specialists (*N* = 6).

**No**.	**Gender**	**Title**	**College type**	**Specialization**	**Region**
1	Female	Professor	Sports college	Kinesiology rehabilitation	Northeast
2	Female	Tutor	Normal college	Sports training	Southwest
3	Female	Associate professor	Normal college	Sports and health	Northwest
4	Male	Tutor	Comprehensive college	Pathology	Northwest
5	Male	Associate professor	Sports college	Sports and health	Southeast
6	Female	Chief physician	Medical college	Sports interventions	Northeast

### 2.2 Interviews

In-depth interviews were conducted guided by six open-ended interview questions (Table 3), which were adapted from previous studies (35, 44). The interview questions were developed based on the research objectives through consultation with three professors in the field of physical health education. These questions aimed to understand the perceptions of sports college students regarding the structure and characteristics of interactive health literacy and explore the difficulties and concerns encountered by them in developing interactive health literacy. The interviews were conducted online and began by building rapport with the participants to ensure that they felt comfortable and willing to share their thoughts and experiences, which was based on previous research (45). Interviews lasted an average of 60 min and were audio-recorded with the participants' consent.

**Table 3:** Interview questions.

**No**.	**Question**
1	In your opinion, how can college students majoring in kinesiology obtain health information? How can one make sense of health information after obtaining it?
2	In your opinion, with whom do college students majoring in kinesiology communicate about the health information they receive? What channels do they use? What skills and abilities are required?
3	What do you think are the common health problems of college students majoring in kinesiology? How can these common health problems be solved?
4	What do you think is healthy for a college student majoring in kinesiology? What is the standard of health for college student majoring in kinesiology? What are the aspects?
5	In your opinion, what are the aspects of healthy living for college students majoring in kinesiology?
6	In your opinion, what are the motivations and confidence in generating healthy behaviors among college students majoring in kinesiology?

### 2.3 Data analysis

The recordings were transcribed verbatim using ROST CM 6 software (46), and the data were stored and managed using Nvivo 14 (47). The data analysis method for the text data obtained from face-to-face offline interviews and social media (e.g., WeChat and Tencent Meeting) online interviews in this study is the same. Data were analyzed using constant comparative analysis and transcripts from each interview were carefully reviewed. After an initial assessment of the data, we analyzed the qualitative data based on the grounded theory, comprising open, spindle, and selective coding. This systematic approach allowed us to identify key themes and patterns in the data (48, 49).

#### 2.3.1 Open coding

Open coding refers to the initial and further conceptualization and categorization of raw empirical data (37). Open coding was used to identify key concepts and categories by capturing key words that appeared in the primary data and using the native words from the empirical data to form free nodes. This was used to develop a theoretical framework for our study.

Step 1: labeling and conceptualization. Through open-ended coding, we identified 288 labels related to interactive health literacy among college students majoring in kinesiology. We then conducted a comparative analysis of these labels to identify key themes and patterns. Guided by the grounded theory, we condensed and refined these labels, resulting in 60 concepts (Tables 4, 5; see Supplementary Tables S1, S2 for detailed data).

**Table 4:** Labeling results.

**Tagged labels**	**Tagged labels**
E1 Own knowledge base of specialized health theories	E145 The need for comprehensive and systematic health knowledge regarding oneself
E2 Self-health is persuasive in communicating health messages	E146 Long-term exercise can be morally constraining
...........	
E142 Staying healthy is the foundation for getting in shape	E286 Nutritional balance to maintain a dynamic balance between energy intake and output
E143 Health is having the energy to go about daily life without getting sick	E287 Endocrine disorders caused by staying up late and eating irregularly
E144 Schools offer health programs to give students access to health information	E288 Invisible sub-health states are unhealthy

**Table 5:** Conceptualization results of 288 tagged labels.

**Number**	**Conceptualization concepts**	**Tagged labels**	**Number of materials**	**Number of participants**
D1	Ability to utilize new media communication tools	4 articles: E78, E131, E187, E244	8	4
D2	Being in good health is persuasive when communicating health messages	2 articles: E2, E186	9	5
D3	Being able to obtain health information from schools	4 articles: E67, E88, E98, E168	18	10
...........				
Total		288 articles	593	349

Step 2: categorization. Using the same continuous comparative analysis process, we further categorized and compared the 60 concepts identified and were able to find that the 60 concepts could be distilled into eight subcategories of “accessing health information, understanding health information, perceiving health functions, shaping health awareness, engaging in health communication, forming health motivation, making healthy decisions and practicing healthy living.” These categories capture key themes and patterns in the data. For example, the category “Obtaining Health Information” contains concepts such as D3, D5, D8, D10, D16, D35, D44, etc. These concepts are related to the sources of students' access to health information, and through continuous comparative analyses, these concepts can be finally refined into the subcategory “C1 Obtaining Health Information” (Table 6; see Supplementary Table S3 for detailed data).

**Table 6:** Results of concept categorization.

**Subsidiary category**	**Concept**
C1 Obtaining Health Information (7)	D3 Can obtain health information from school D5 Can obtain health information from people around them D8 Can obtain health-related information on the Internet D10 Can obtain health information through social practice experience D16 Can strengthen the mastery of health information and improve health literacy through examinations D35 Can obtain health information from WeChat public platforms D44 Focus on health information related to oneself
......	
C8 Practicing Healthy Living (9)	D23 Healthy living requires the adoption of behavioral habits such as diet, rest, and exercise D29 Long-term exercise habits can improve physical fitness and prevent chronic diseases D30 Healthy living habits and exercise behaviors require strong self-discipline D31 Pre-exercise warm-up and post-exercise stretching are effective in preventing sports injuries D43 Ability to acquire knowledge of health-promoting exercises D50 Regular participation in physical activities can improve social interaction skills D52 Health management requires focus on diet and food combinations D53 Dynamic balance of energy intake and output can promote health D54 Maintaining a regular routine and not staying up late

#### 2.3.2 Axial coding

Axial coding involves categorizing and comparing various concepts or categories by disassembling and reorganizing the free nodes to extract the main concepts or categories (50). In spindle coding, we disassembled and reorganized the free nodes, enabling the systematic and comprehensive analysis and conceptualization of the data.

Through further comparative analysis, we reduced the subcategories to three main categories: harnessing health information, promoting health interactions, and addressing health problems (Table 7).

**Table 7:** Results of the main axis coding for eight categories.

**Sub-category**	**Subsidiary category**
B1 Harnessing Health Information	C1 Obtaining Health Information C2 Comprehending Health Information
B2 Promoting Health Interactions	C3 Perceiving Health Functions C4 Shaping Health Awareness C5 Participating in Health Communication
B3 Addressing Health Problems	C6 Forming Health Motivation C7 Making Healthy Decisions C8 Practicing Healthy Living

#### 2.3.3 Selective coding

Selective coding is the process of analyzing the connections between key concepts or categories and making ongoing comparisons to identify the core categories (37). Selective coding is the final stage of the grounded theory analysis process (51, 52). We identified the following core category: interactive health literacy among college students majoring in kinesiology in China. This core category integrated all of the other categories, concepts, and labels identified during the coding process, providing a comprehensive, holistic view of the research question.

### 2.4 Trustworthiness

Theoretical saturation, guided by the grounded theory, is related to plausibility (37). Several tests and checks were conducted to ensure the reliability and validity of the coding process (53, 54). First, we conducted a coding consistency test to reduce subjectivity and increase confidence in the coding results. We randomly selected five profiles from the raw data and asked two research assistants familiar with the use of the Nvivo 14 software to simultaneously code the samples back-to-back. Subsequently, we calculated the coding repetition rate of the sample and compared it to the results of the initial coding. In addition, we conducted a theoretical saturation test using the remaining five interviews as a reference to determine whether new categories and relationships could be identified and whether the theoretical model reached saturation. These results demonstrated the reliability and validity of our coding process and the resulting theoretical framework.

## 3 Results

**Figure 1:**
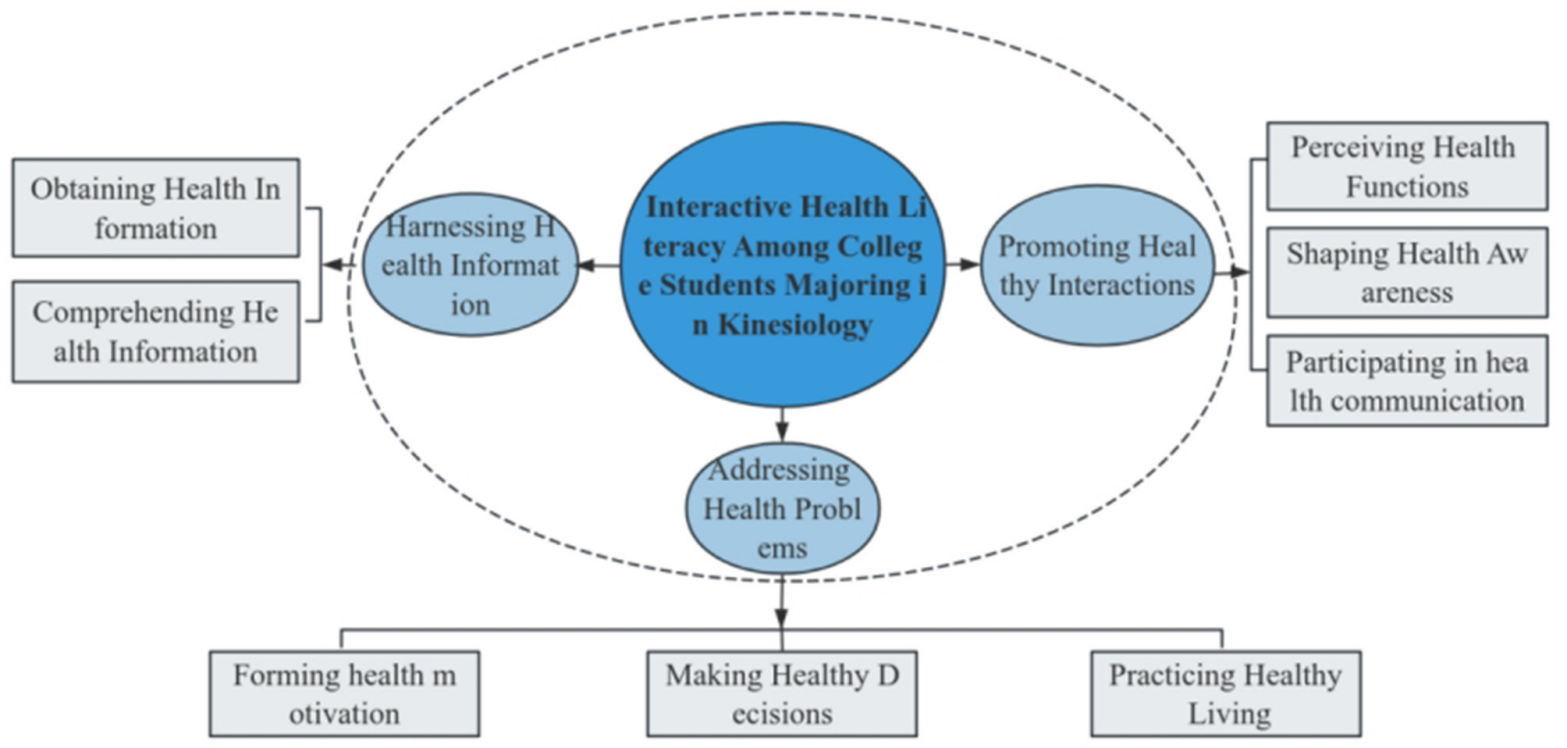
Interactive health literacy among college students majoring in kinesiology.

The data analysis identified three major themes and several subthemes that reflect aspects of interactive health literacy among college students majoring in kinesiology (Figure 1).

### 3.1 Harnessing health information

Harnessing health information refers to the ability to access and understand health information and independently acquire knowledge through health education. This category describes specific behaviors in acquiring health information.

#### 3.1.1 Obtaining health information

Obtaining health information refers to the main sources and channels for acquiring health information as well as the associated skills. Many interviewees stated that health education in college was their main source of health information, followed by various social channels and online information.

*I think health courses in school and guidance from teachers are the most important sources of health information for college students majoring in kinesiology. Moreover, this guarantees the authority and correctness of the health information obtained*. (Participant 1)

College students majoring in kinesiology filter the health information they obtain and search for health information related to themselves through the Internet and other means. Considering their professional attributes, college students majoring in kinesiology often experience contact with the outside world during college, providing opportunities to obtain health information. For instance, some interviewees mentioned that when they worked part-time at a gym, they could formulate suitable fitness programs based on the health conditions of various groups of people to enhance their physical fitness. This helped them learn health information.

*After learning this information, we can consolidate its use in practice by giving customers in need a suitable fitness program based on their lifestyles so that they can maintain healthy habits and feel it themselves in practice*. (Participant 3)

#### 3.1.2 Comprehending health information

The participants stated that after acquiring health information, they needed to understand and internalize it. This provided the foundation for subsequent communication and dissemination of health information.

The participants had different ways of understanding health information due to differences in cognition and understanding and based on their individual health status. For instance, many interviewees stated that physical activity and health promotion were relevant; thus, health-related information could be understood in the context of their professional strengths. Learning to prevent sports injuries during exercise and obtaining skills to deal with injuries in an emergency promotes good health. Exercise can help alleviate and prevent the occurrence of chronic diseases, which is important for maintaining healthy habits in daily life.

*Kinesiology students accumulate experience in the process of regular classes and during exercise. These experiences promote health knowledge, and knowledge changes their behavior to maintain positive life habits. For example, some sports injuries that occur during exercise motivate people to learn more and help themselves understand this information in the context of a real exercise situation*. (Participant 2)

Information on the Internet is not always accurate. College students need to have the ability to identify accurate health information. For students majoring in kinesiology, health education in college is the main channel to obtain health information, and they can understand this information through teachers' explanations. When encountering health information that is difficult to understand, they can consult professionals for advice.

*First, students can carefully read health instructions in books. If students encounter areas that are difficult to understand, they can consult teachers or professionals for their opinions on these issues. Second, students usually share this health information with coaches or professors in specialized classes and communicate with them to verify the accuracy of this information*. (Participant 3)

### 3.2 Promoting healthy interactions

Promoting healthy interactions reflects individuals' communication and interaction on health issues.

#### 3.2.1 Perceiving health functions

Communicating about health requires understanding health and its benefits. Most participants stated that health includes physical and mental health and that different states of health have different effects. For instance, they believe that physical health is the basic state of life, that mental health problems affect physical health, and that physical exercise can alleviate mental stress and enhance physical fitness to achieve a healthy life.

*I believe that physical health means that there is no disease that affects normal life, and that all indicators are within a reasonable range. On the basis of physical health, we can maintain a happy mood. The lack of mental illnesses indicates mental health. Being able to understand what health is and the importance of health is the only way to maintain good lifestyle habits and stay healthy*. (Participant 5)

#### 3.2.2 Shaping health awareness

Understanding the function of health shaped personal health consciousness and enhanced perceived importance of health among the participants. In recent years, the physical quality of college students has declined, which has caused concern. Colleges can use a variety of methods to provide health education and shape students' health consciousness. For instance, health education courses could show health warnings to attract student attention. Among after-school activities, student organizations could promote health education, disease prevention, and knowledge of medication to help students realize the importance of health, thereby shaping healthy lifestyle habits. Physical exercise can enhance physical fitness and motor skills and strengthen social skills through interaction with people during exercise. Long-term maintenance of healthy lifestyle habits can improve health self-confidence and motivate individuals to engage in healthy behaviors, thereby improving health awareness.

*The resistance of kinesiology college students is relatively good, which leads to common illnesses due to bad lifestyle habits. For example, kinesiology college students stay up late, smoke, drink, and don't wear jackets after sweating during exercise. These actions affect their health. Schools can organize health education activities to show situations relevant to students to help them resist bad habits and focus on their health*. (Participant 3)

*Nowadays, the physical fitness of college students is declining; they can't even finish the basic physical test, and they faint while running. People who see this phenomenon consciously do sports to enhance their physical fitness, mainly for the sake of their own health*. (Participant 6)

#### 3.2.3 Participating in health communication

Participating in health communication reflects the factors for health communication and reasons for active participation and is the most important subcategory of promoting healthy interactions. The participants stated that communicating health information requires a variety of competencies, as well as the methods and channels of communication. They noted that with the development of new media, the use of communication tools is important.

*Dissemination of health information is a process of communicating with people that requires good communication skills. There are certain requirements for the linguistic ability of the person who transmits the information to ensure the accuracy of the expression. Sometimes a difference in one word can cause the dissemination of incorrect health information*. (Participant 5)

*Nowadays, college students pay more attention to information on the Internet and get a lot of information from the Internet, so they also use some new media tools when disseminating health information. For example, making short videos with explanations is easy for people to understand. If they can operate a public health number, they can reach more people and deliver health information faster*. (Participant 6)

Individuals with good health are persuasive in communicating health information, provide health advice based on their own practical experience, and take the initiative to share and exchange health information with others when encountering health problems. This reflects health interaction.

*I feel that college student majoring in kinesiology need to make sure that they are healthy when sharing health information. They are not only responsible for their own health but also need to obtain health through their own physical skills. People initiate communication with others to solve their own health issues*. (Participant 7)

### 3.3 Addressing health problems

Addressing health problems reflects the ability to make health decisions and use health knowledge and available health information to change one's health.

#### 3.3.1 Forming health motivation

Health motivation can form independently through the acquisition of health information and knowledge. Confidence and internal needs conducive to health behaviors create various types of health motivation. Different health motivations lead to different health needs and lifestyles; however, the ultimate goal is to maintain health.

*Physical exercise can improve one's body shape, athletic ability, and physical function, ultimately maintaining health and preventing common diseases, This is the main purpose of exercise. Participating in different sports causes different stress responses to improve physical functions*. (Participant 9)

*I think there are different purposes for college students to participate in sports; some people do it to stay in good shape, some people do it to reach their personal sports goals, some people do it because they love sports, some people do it to keep their body healthy, and some people do it to satisfy their self-esteem*. (Participant 8)

#### 3.3.2 Making health decisions

Making health decisions refers to identifying healthy lifestyle habits and engaging in health-promoting behaviors based on knowledge.

*Nowadays, many students do not have good dietary habits, such as skipping breakfast, irregular meal times, eating late-night snacks, and other situations. They also have irregular work and rest habits. We need to pay attention to this situation because it can have a negative impact on health*. (Participant 11)

*Adequate warm-up preparatory activities before physical exercise can effectively prevent sports injuries, and accumulated sports experience enables students to pay attention during exercise*. (Participant 15)

#### 3.3.3 Practicing healthy living

Practicing healthy living is the ultimate expression of interactive health literacy, which manifests in positive lifestyle habits. Long-term exercise habits enhance physical fitness, prevent chronic diseases, enhance social interaction skills, and maintain mental health. A good diet and work habits are required to keep individuals healthy.

*Among lifestyle habits, first, a healthy diet, including avoiding overeating and anorexia, is required to maintain a balance between energy intake and output. Second, regular work and rest, having enough sleep, and not often staying up late can relieve stress. This, combined with a scientific exercise program, can maintain a healthy lifestyle*. (Participant 13)

*The hygiene and healthcare habits of college students majoring in kinesiology are not very good. They focus on their personal image but do not pay attention to the living environment. All these factors affect their health*. (Participant 11)

*I think that sports activities are social in nature, and if you participate in sports activities regularly, you will surely interact with others. You can also improve your ability to socialize with others*. (Participant 12)

## 4 Discussion

This study investigated the interactive health literacy of college students majoring in kinesiology. The results identified students' health and health problems, relationships between physical activity and interactive health literacy, and health education required in colleges to improve interactive health literacy among college students majoring in kinesiology.

### 4.1 Interactions between dimensions

The interactions between harnessing health information mastery, promoting healthy interactions, and addressing health problem are complex and multifaceted. Harnessing health information is the foundation of the other two dimensions. Promoting healthy interactions is a direct transition from harnessing health information to addressing health problems. Addressing health problems dimension is the final manifestation of the other two dimensions.

In interactive health literacy, the relationship between harnessing health information and addressing health problems is more important. Grasping health information is the basic factors of harnessing health information among college students majoring in kinesiology and affects the development of healthy habits in their daily life and their ability to solve health problems. College students who can harness health information have good health awareness and engage in health-promoting behaviors in their studies and life, thus maintaining healthy lifestyle habits and solving the health problems they encounter. Similarly, health promotion can help college students majoring in kinesiology solve health problems. Promoting healthy interactions involves an in-depth understanding and application of health information, which helps skillfully solve various health problems that may be encountered.

### 4.2 China's health requirements

The results of this study indicate that the interactive health literacy of college students majoring in kinesiology can play a role in China's strategy for a healthy nation. Sports support the implementation of the “Healthy China” strategy, which not only highlights the role and value of sports but also demonstrates the nature of sports as being closely related to life. Health literacy helps effectively promote healthy behaviors among college students through sports activities and achieve the goal of population's health literacy. Through health education in colleges, students majoring in kinesiology can actively seek health knowledge and implement it to harness health information. This improves their ability to solve health problems, their communication skills, and their ability to make health decisions. Moreover, this generates motivation and confidence regarding healthy behaviors, ultimately improving health.

Students should not only learn the theoretical and technical knowledge of kinesiology but also master the related health knowledge through health education to improve their health literacy and skills, health self-management abilities, and participation in public health affairs. Interactive health literacy reflects the ability to acquire and apply health information. Health literacy is required for students majoring in kinesiology in future dissemination of health knowledge, including health promotion and education. Moreover, health literacy is necessary to formulate scientific, feasible, and effective health behavior programs.

The main channels and sources for students majoring in kinesiology to acquire and understand health information are through health education in colleges, with off-campus experiences being a supplementary channel. Colleges provide health education to students based on their kinesiology majors, and students majoring in kinesiology grasp relevant health information based on their majors and verify it in practice. For instance, sports injuries can increase students' health knowledge of preventing sports injuries, as well as the subsequent maintenance and medication information, which can be utilized in daily life. This health information is frequently accessed by college students majoring in kinesiology, which is a unique professional characteristic that enhances their harnessing of health information. Students acquire the relevant health information in sports practice and utilize their professional strengths in life to understand the acquired health information by using their training experience. Therefore, students majoring in kinesiology, due to their unique professional advantages, can guide people to understand health, thereby increasing health literacy.

### 4.3 The basis of structural models

The structural model of interactive health literacy among college students majoring in kinesiology is an improvement on related scientific theories, including the knowledge, belief, and action theory (KBAP); trans theoretical model; and social cognitive theory. Moreover, this model is an improvement on related scientific theories, including the knowledge, belief, and action theory (KBAP); trans theoretical model; and social cognitive theory. These theories fit the connotation of the interactive health literacy system for college students majoring in kinesiology. Furthermore, constructing a system of interactive health literacy among college students majoring in kinesiology combines the social cognitive theory and health concepts of proactive health and treating the future disease in Chinese medicine.

The KBAP is a model of acquiring health knowledge, generating health beliefs, and forming health behaviors. It explains that individuals need to go through three stages to change their health behaviors and that individuals can gradually build up their beliefs only when they acquire health-related knowledge, think deeply about it, and have a clear sense of responsibility. College students majoring in kinesiology should learn this theory through health education to develop healthy lifestyle habits, obtain health knowledge, and generate health beliefs.

The trans theoretical model, also known as the behavioral staged transformation theoretical model, is widely used in research on health behavior change, which seeks to elucidate the process by which behavioral change occurs, rather than merely why it occurs. The model depicts individuals at different stages of adverse behavior change by adopting appropriate behavioral transformation strategies, ultimately acquiring positive behaviors and being able to sustain them. The trans theoretical model suggests that college students majoring in kinesiology gradually develop positive health behaviors through health education in college, maintain these behaviors after achieving good health literacy, and positively influence others by creating their own good health image. This promotes the dissemination of health behaviors and information.

The concept of proactive health was first proposed in 2015 in China. The concept emphasizes increasing the micro-complexity of the human body by actively applying controlled stimuli, prompting the human body to adapt to various changes and identify and assess its own state and direction and degree of development. This helps individuals choose appropriate lifestyle factors and actively and positively use medical methods to promote the development of healthy behaviors. Moreover, this promotes adaptive self-regulation to improve physical function, prevent diseases, and maintain a healthy state. The term “treating the future disease” in Chinese medicine first appeared in the Yellow Emperor's Classic of Internal Medicine. It emphasizes that when the human body is healthy, we should focus on healthcare methods to strengthen the body, maintain health, improve quality of life, and prevent diseases.

Establishing a structure system of interactive health literacy among college students majoring in kinesiology would help students clearly understand their health and adopt behaviors such as health promotion and management. As the Healthy China Initiative continues to advance, proactive health will gradually become an area of focus in addition to medical and healthcare reform. Exercise science is an important component of proactive health and will become an important part of the future medical system with increased responsibilities and development opportunities.

### 4.4 Practical paths to improve health literacy among Chinese college students

What kind of health education should be carried out in Chinese colleges and universities in order to improve the health literacy level of college students, and the relevant educational institutions and governmental departments should provide the key human and material support system from various aspects, and the specific implementation suggestions can be based on the three interactive theoretical models of “harnessing health information, promoting health interaction and addressing health problems” constructed in this study. The specific implementation suggestions can be based on the theoretical model of the three interactions of “mastering health information, promoting health interaction and solving health problems” constructed in this study. Through the following measures, we can systematically improve the comprehensive literacy of sports majors in health information processing, interactive participation and health problem solving, and ultimately achieve the positive cycle of “information input—interactive reinforcement—practical internalization.”

(1) Optimize the ability to acquire and understand health information. The combination of health theory and practice acquired by students in the classroom can promote the enhancement of the acquisition and comprehension of health information. Therefore, it is necessary to optimize and improve the theoretical courses of health education in which students acquire health knowledge, as well as the practical courses in which health information is applied. Firstly, schools can offer integrated health courses, embedding modules such as Exercise and Health and Physical Fitness Assessment and Promotion in physical education courses, and strengthening the acquisition and comprehension of health information through case studies (e.g., prevention of sports injuries and nutritional matching). For example, basketball teaching is integrated into the exercise physiology curriculum in colleges and universities to enable students to understand the correlation between exercise intensity and health indicators (55). Secondly, build a digital health information platform, develop a health knowledge database (e.g., exercise prescription library, chronic and management guide), and push personal learning resources in combination with AI to improve the efficiency of information acquisition.
(2) Strengthening the interactive health participation mechanism. The promotion of health literacy requires multi-party collaboration, with the establishment of a “four-in-one” interactive network that links medical institutions, schools, communities and social organizations to carry out activities such as health lectures and physical fitness tests. For example, colleges and hospitals are working together to offer “exercise rehabilitation workshops,” where students learn how to solve health problems through practice. Some studies have shown that the frequency of communication among students participating in health-themed clubs has increased by 40 percent, and the information for health decision-making has been significantly enhanced. Based on this, university sports clubs are expanding their functions, incorporating the cultivation of health literacy into their assessment indicators, and requiring them to organize regular health-themed debates, first-aid skills competitions, and other activities.
(3) Deepening practical measures to solve health problems. To improve students' health literacy, we implement a closed-loop management process of “health profile-feedback,” establish students' health literacy e-portfolio, embed real health problem-solving scenarios in physical education, design situational health challenge tasks (56), and dynamically track students' health behavior (e.g., frequency of exercise, quality of sleep) to generate improvement suggestions through algorithms according to the specific situation of students. The algorithm generates improvement suggestions for students' specific situations.

## 5 Conclusions

This study developed a theoretical model of interactive health literacy among college students majoring in kinesiology. We identified three main categories, eight subcategories, 60 concepts, and 288 labels. The system theory passed the theory saturation test and coding consistency test, which demonstrated good reliability.

The results of this study can be used to evaluate interactive health literacy among college students measuring in kinesiology. Our findings can be used to identify problems in college health education and provide a reference for developing health education programs and strategies to address these problems. This study can help colleges, universities, and related administrative departments assess the interactive health literacy of college students majoring in kinesiology and determine strategies to increase their health literacy. Interactive health literacy is a cognitive ability. College students must acquire relevant health knowledge and deeply consider it to improve their health literacy. Promoting healthy interactions plays an important role in the health communication of college students and can cultivate their communication abilities and use of new media communication tools. Practicing healthy living refers to the ability to make healthy decisions and is an advanced cognitive and social skill that can help students maintain healthy lifestyle habits.

This study constructed an interactive health literacy structural model for Chinese sport major college students based on the Grounded theory. Although the model may face the challenge of cultural or educational differences in cross-national applications, its core framework still has replicable potential, and its strengths and weaknesses exist simultaneously. Its strengths are reflected in: (1) Methodological strengths: adopting a bottom-up inductive research path rooted in theory, the model is constructed in an original and systematic way through in-depth interviews and coding analyses, explaining the unique health behavior of university students majoring in physical education under the Healthy China Strategy. During the research process, the participants' cognitive maps were gradually improved through continuous comparison and theoretical saturation testing, and the rooted theories are highly replicable, so that the same methodology can be used to construct theoretical models of the health literacy of students in their own countries according to different national conditions. (2) Theoretical universality: The three dimensions of “harnessing health information,” “promoting healthy interactions” and “addressing health problems” in the model are consistent with the WHO health literacy principles of “access-understand-apply” and are applicable to different cultures. The cross-cultural commonality of social interaction has been verified in cross-disciplinary thematic learning studies, such as the peer support effect in western sports communities. (3) Room for local adaptation: in cross-national applications, countries can make local adjustments according to the theoretical framework. In terms of content, the health knowledge in the model can be replaced with local health knowledge (e.g., replacing Tai Chi with yoga); in terms of policy synergy, local education policies (e.g., the European Union's Healthy Schools Initiative) (57) can be combined to optimize the implementation of the specific pathways of improving health literacy.

Its disadvantages are: (1) Rooted theory requires the investment of sufficient time, energy and data resources, and the researcher needs to collect, analyse and interpret a large amount of data in order to facilitate the construction of a theoretical model based on participants' experiences. In the process of cross-cultural research, language barriers or cultural differences can pose a great challenge to the researcher and can affect the process of data collection and analysis. (2) Applying the model according to cultural differences in different countries: firstly, in terms of health information acquisition and understanding, Chinese students rely on school health education curricula and official platforms and are more influenced by local health knowledge such as traditional Chinese medicine and health maintenance; whereas Western countries prefer to acquire health information from social media and community resources and advocate scientific knowledge of sports medicine and sports nutrition. Second, in terms of health interaction patterns, China emphasizes collectivist community activities and teacher-led health knowledge transfer in health education, whereas Western countries focus more on individual exercise communities, such as the gym personal training group model, and place more emphasis on student self-directed exploratory learning, such as health interactions in project-based learning. Finally, when it comes to solving health problems, Chinese students tend to receive health guidance from their families and teachers, while Western cultures encourage students to make independent health decisions. (3) Differences in the educational systems of different countries: in this study, Chinese physical education curricula are usually separated from health literacy, whereas in Western countries, health literacy may be organically integrated into interdisciplinary programs, and assessment of students' health literacy levels is more concerned with critical thinking. Therefore, although the interactive health literacy structure model constructed in this study can cover the health literacy structure of most students in physical education, there are some potential limitations in the process of cross-national application. Researchers need to take these limitations into account when conducting cross-national applications to ensure the rigor of the data collection and analysis process so that it is appropriate for the research questions to be addressed.

In an era of focusing on health for the entire population, specific manifestations of interactive health literacy among college students majoring in kinesiology must be understood to promote health literacy and develop interactive health literacy programs. This study provides insights into the interactive health literacy among college students majoring in kinesiology and suggests that future studies should validate and extend these findings. By examining the specifics of interactive health literacy among college students majoring in kinesiology in different regional institutions, we can gain a comprehensive understanding of the opportunities and challenges for developing population health literacy for all. These findings can help develop effective health education strategies and universal health education programs to enhance health literacy and promote national health development.

## Data Availability

The datasets presented in this study can be found in online repositories. The names of the repository/repositories and accession number(s) can be found in the article/ Supplementary material.
