# Supplementary material for: Developing a model of interactive health literacy among college students majoring in kinesiology: a grounded theory approach
Source: Front Public Health. 2025 Apr 30;13:1510035. doi: 10.3389/fpubh.2025.1510035 (PMC12075114; doi:10.3389/fpubh.2025.1510035)
Supplement: Supplementary file 1 [file Table_1.docx]

**Table S1.** Labeling results

| **Tagged Labels** | **Tagged Labels** |
| --- | --- |
| E1 Own knowledge base of specialized health theories | E145 The need for comprehensive and systematic health knowledge regarding oneself |
| E2 Self-health is persuasive in communicating health messages | E146 Long-term exercise can be morally constraining |
| E3 Self-discipline promotes health and exercise behaviors | E147 Unhealthy eating habits cause hyperuricemia |
| E4 Knowing health information but having a hard time maintaining healthy behaviors | E148 Exercise promotes bone density and bone mass growth |
| E5 Targeted exercise for physical weakness | E149 Developing good physical activity habits is an important part of the health message |
| E6 Awareness and skills in disease prevention and management | E150 School programs tend to focus on strength and speed training with little endurance training |
| E7 Reasonable time for healthy exercise | E151 Intervening with medications for common diseases |
| E8 Exercise at least three times per week | E152 Regular exercise can prevent obesity and other diseases |
| E9 Exercise can help with addiction | E153 The human body is in a dynamic process of health; health is having a stable health condition over a long period of time |
| E10 Exercise reduces anxiety and promotes sleep | E154 Gaining health knowledge from part-time experience |
| E11 Exercise can change body structure and mental outlook | E155 Kinesiology students are emotionally stable, resilient, and socially adaptable |
| E12 Exercise can be good for the heart | E156 Nutritional rationing is a higher pursuit of dietary health, and not caring falls within the scope of health |
| E13 Exercise can regulate mood and relieve stress | E157 CPR and other skills needed for lifeguard certification |
| E14 Venting repressed emotions promotes good health | E158 Physical activity is associated with self-regulation |
| E15 Seeking help from a teacher or professional to understand health information for yourself | E159 Shift from a treatment-centered approach to proactive prevention and greater health literacy |
| E16 Obtaining health information from families | E160 Mental health is the absence of mental illness |
| E17 Site-specific, equipment, and warm-ups can prevent sports injuries | E161 Depression, anxiety, and schizophrenia are mental illnesses |
| E18 Communicating health information requires good presentation skills | E162 Fewer mental illnesses among college students majoring in kinesiology |
| E19 Paying attention to health information that is relevant to you | E163 Women have better habits than men |
| E20 Exchanging health information with fitness trainer to verify correctness | E164 Relative paucity of specialized health information that schools can provide |
| E21 Exchanging workout information with athletes | E165 Kinesiology students who study nutrition have control over their diets |
| E22 Sharing health information with people with physical illnesses | E166 Physical activity behaviors are not intended to promote health |
| E23 Passing on health knowledge to students; maintaining good living habits | E167 Health is having the energy to meet the basic needs of life, education, and work |
| E24 Sharing and exchanging health information with family and friends | E168 Students majoring in sports and human biology are knowledgeable about health information |
| E25 Recreational exercise to stay healthy; professional exercise to improve physical fitness | E169 Good physical fitness and no physical disabilities |
| E26 Having good interpersonal relationships and a positive outlook is considered mental health | E170 Students who do not exercise because they want to be healthy |
| E27 Monitoring eating time | E171 Regular exercise is 30 minutes of moderate-intensity exercise three times per week |
| E28 Poor diet can easily lead to obesity | E172 Health knowledge gained from own practical experience |
| E29 Self-discipline to be able to keep exercising | E173 Poor lifestyle habits of kinesiology students lead to illness |
| E30 Asking family members, teachers, and classmates about their health problems | E174 Physical exercise leads to better physical fitness, thus satisfying one’s vanity |
| E31 School health program lectures to promote health communication | E175 Taking the time to read the information to ensure that health knowledge is correct |
| E32 Dissemination of health information is as accurate as possible | E176 Training in safety and emergency first aid should be mandatory |
| E33 Autism and depression are both mental health issues | E177 Communication skills are important to make others want to listen to you |
| E34 Behavioral and verbal expressions are more natural | E178 Two days of recovery is required after moderate- and high-intensity exercise |
| E35 Learning to relax your mind and resting properly to stay healthy | E179 Not overeating or having anorexia, and having regular eating habits is part of a healthy lifestyle |
| E36 Determining mental health through psychological testing scales | E180 Going to bed by 11:30 p.m. |
| E37 Delivering health messages to substitute students | E181 Health includes physical health, mental health, The meaning of this is unclear. Please clarify what you mean by this. |
| E38 Ability to present your ideas politely | E182 People play sports if they want to be healthy |
| E39 Sharing the workout information in WeChat | E183 Kinesiology students are physically fit for emergency first aid work |
| E40 Online fitness bloggers share health information | E184 Having health knowledge and understanding heath concepts, but not engaging in healthy behaviors |
| E41 External language stimulates self-involvement in movement | E185 People who want to build muscle focus on their diet |
| E42 Peers share health information with each other | E186 Work related to the need to ensure their own health and thus the health of others |
| E43 Intentional health maintenance through the study of specialized courses | E187 Ability to run a public website |
| E44 Awareness of keeping fit through exercise | E188 Gain relevant experience from one’s own practical experience |
| E45 Visualization of external devices to be able to observe one’s own health at any time | E189 Regular exercise promotes moral health |
| E46 Practice examples can help understand health information | E190 Health includes mental health, physical health, and a certain degree of social adaptability |
| E47 Differences in autoimmunity lead to different body types | E191 Students have less health information and are less health literate |
| E48 Training and exercise experiences lead to a better understanding of health | E192 Supervising the acquisition of health information through examinations |
| E49 The social behavior of exercise relieves psychological stress | E193 Good exercise habits promote good health |
| E50 Knowledge of chronic disease prevention through sport | E194 Athletic skills to meet the requirements of the kinesiology program |
| E51 Kinesiology students have strong athletic and physical abilities | E195 Ability to manage simple epidermal sports injuries |
| E52 Psychological learning stress among kinesiology students | E196 Having a good external physique |
| E53 Kinesiology students have poor hygiene practices | E197 Ensuring the authenticity of health knowledge |
| E54 Better physical indicators for kinesiology students | E198 Getting sufficient sleep, not going to bed late, and going to bed after 11:00 p.m. |
| E55 Kinesiology students are in better spirits | E199 Habits are more important than self-control |
| E56 Kinesiology reinforces social behavior | E200 Rejuvenation of the three health problems |
| E57 Cultivating hard-working qualities in physical exercise | E201 Kinesiology students are interested in sports |
| E58 Theoretical knowledge of sports can guide athletic behavior | E202 Know more about health-related professions, others just basic understanding |
| E59 Theoretical knowledge of sports combined with practice helps achieve good workout results | E203 People who are concerned about their health take the time to consult the literature |
| E60 A love of physical activity enables consistent exercise | E204 Obtaining health information online is easy |
| E61 Psychological regulation through sports | E205 Ability to recognize health information and access relevant articles |
| E62 Physical activity promotes mental health | E206 Healthy habits to maintain weekly exercise |
| E63 Being active with regular physical activity | E207 The secretion of hormones during exercise causes psychological pleasure |
| E64 Participation in sports improves ability to interact with others | E208 Students exercise alone at the gym or participate in instructor training |
| E65 Improvement in self-resistance and immunity to prevent diseases | E209 College students do not understand healthy habits |
| E66 Adequate sleep helps maintain a good physical condition | E210 Students must have knowledge and skills in first aid, and schools should organize competitions |
| E67 Obtaining health knowledge from books, papers or the internet | E211 Health knowledge is shared through self-publishing |
| E68 Exercise in moderation; excessive exercise can interfere with normal life | E212 Swimming regulates the external body temperature and strengthens the immune system. |
| E69 Practical health cases help better understand | E213 Being physically active makes one mentally tough |
| E70 Health problems are mainly physical | E214 The problem of skipping breakfast and binge snacking |
| E71 Knowledge of anatomy and physiology | E215 Using exams to promote health literacy among students |
| E72 Physical health as evidenced by indicators within normal limits | E216 Knowledge of acute management and chronic phase stretching in sports rehabilitation |
| E73 Good communicating skills, outgoing personality, lively, and cheerful | E217 Chronic injuries or sports illnesses caused by exercise |
| E74 A regular routine is necessary for good health | E218 Classroom theoretical knowledge combined with one’s practical experience in determining sports injuries |
| E75 Warm-up and relaxation activities help prevent sports injuries | E219 A good exercise system promotes the proper functioning of other systems |
| E76 Passionate about sports and exercise | E220 Retweeting and sharing health information on the platform |
| E77 Critical thinking and ability to determine if health information is correct | E221 Checking the back of the wrist while playing tennis can prevent injuries |
| E78 Ability to record and forward health media materials | E222 College students’ physical fitness declines, and myopia is a serious problem |
| E79 Preparation of essential medications for diseases related to you | E223 Access to a wide range of health information from the social dimension |
| E80 Pharmacologic prevention of simple physical ailments | E224 Using mobile applications to promote understanding of health literacy |
| E81 Health is having no serious physical injuries | E225 Dissemination of health information to social service recipients |
| E82 Health is the absence of disease | E226 Getting a good night’s rest and maintaining daily energy |
| E83 Chronic diseases are common | E227 Becoming aware of one’s health |
| E84 Low body fat percentage and lots of muscle mass | E228 Chronic diseases, such as diabetes, affect health |
| E85 Good body shape and form | E229 Preventing chronic diseases through exercise |
| E86 The standard of health is to have good body form and functioning | E230 Maintaining muscle mass and striving for a pleasant appearance rather than for health |
| E87 Have a basic understanding of other people’s habits and behaviors | E231 Reducing fractures after an accidental fall |
| E88 Acquiring health knowledge in classrooms and textbooks | E232 Rehabilitation and emergency management after sports injuries are health behaviors |
| E89 Understanding the science of exercise | E233 Work part-time at a gym or via the Internet |
| E90 Scientific exercise requires specialized theoretical knowledge | E234 Applying health knowledge and learning in volunteer activities |
| E91 Resilient to stress and able to alleviate bad moods | E235 College students need to be able to recognize true and false information |
| E92 Knowledgeable about sports nutrition knows what to do | E236 Public lectures and popular science are good avenues |
| E93 Ability to help others with related health issues | E237 Exercise 150–300 minutes per week, with at least 25 minutes of moderate to high intensity exercise per session |
| E94 Seeking medical attention for serious health problems | E238 Kinesiology students have good social interaction skills |
| E95 Health problems associated with a sedentary lifestyle | E239 Middle-aged and older patients with underlying diseases maintain a basic level of exercise that is not strenuous |
| E96 Aggressive or competitive | E240 Improving health and preventing disease through exercise |
| E97 Regular physical activity reduces depression | E241 Availability of some after-school hours |
| E98 Teacher-led student exposure to health-related knowledge in schools | E242 Availability of the means and channels of transmission |
| E99 Anxiety negatively affects quality of life | E243 Not stretching after exercise with wrong movements |
| E100 The authority and logic of health knowledge can ensure that sharing health knowledge | E244 Academic literacy in writing and editing images |
| E101 Health and sports are highly relevant and easy to understand | E245 Online knowledge requires judging right and wrong, and one’s own experience helps others |
| E102 Health is simply the harmonization of mind and body | E246 Being able to live with a chronic disease for a long period of time is also a healthy condition |
| E103 Daily health behaviors include physical activity, eating, and resting | E247 Excessive use of electronic devices produces myopia and cervical spine problems |
| E104 Health means no interference with normal activities and exercise and not feeling pain | E248 The presence of a long-term hidden disease is not a state of health |
| E105 Eating regular meals, living a regular lifestyle, and being active | E249 Inadequate capacity of university students to take care of themselves |
| E106 Maintaining your exercise routine; just sweat it out | E250 Traditional health means the absence of disease |
| E107 Little attention is paid to the issue of disease prevention | E251 Schools provide health interventions for students |
| E108 Obtaining health information through short Internet videos | E252 Mental health refers to mindsets and attitudes exhibited by individuals |
| E109 Eating a sensible diet with a balanced energy intake | E253 Working people have little time to exercise, 3-4 times per week |
| E110 Following health information that interests you | E254 Patients with underlying diseases are not perfectly healthy |
| E111 Paying attention to your diet according to your exercise habits | E255 The difference between optimism and pessimism |
| E112 Developing training content based on the training program | E256 Sleeping late interferes with breakfast, which is unhealthy |
| E113 Health risks associated with high-intensity exercise | E257 Not being well informed about medication use can cause medication abuse or misuse |
| E114 Taking drugs to build muscle | E258 Health includes physiological, physical, and mental health |
| E115 Physical training combined with sport-specific skill training | E259 Mastering standard movements without forcing oneself to complete them |
| E116 Being sociable and having good interpersonal relationships is considered mental health | E260 Ensuring one’s health literacy and disseminating the right information |
| E117 Being conscious of your body image | E261 Mental health reflects on physical health |
| E118 The quest for better results and perfect athletic performance | E262 Rest is more important than diet, and adequate sleep is different for everyone |
| E119 Lack of vigilance against infectious diseases | E263 The authority of the identity can be recognized by more people |
| E120 Staying healthy from disease | E264 Eating smaller meals to ensure a healthy diet |
| E121 Developing a sensible training program | E265 Health is being comfortable |
| E122 The atmosphere of the workout environment influences workout habits | E266 Sports, muscle, or joint injuries |
| E123 Difference between exercise and training; exercise is related to health | E267 Cultivating students with authority in the classroom to lead the way |
| E124 Regular physical activity leads to a strong body and promotes health | E268 Active rehabilitation after sports injuries |
| E125 Resistance prevents disease | E269 Chronic unhealthy habits can cause changes in the body |
| E126 Playing ball to build arm strength | E270 Physical health, mental health, and health related to interpersonal interactions |
| E127 Obtaining knowledge of sports injury prevention from classmates | E271 Not realizing the importance of health when not injured, need more cases |
| E128 Obtaining health information from health magazines | E272 Sensing relevant health information through one’s situation |
| E129 Infectious disease problems unhealthy sex | E273 Meeting the expectations or approval of others affects self-esteem |
| E130 When people need to know something, they tell them | E274 Developed Internet, more experts, confusing information |
| E131 Ability to use communication tools | E275 Different intensities and types of exercise can improve different diseases |
| E132 Smoking, drinking, and bad habits | E276 Exercise promotes the body’s indicators |
| E133 Knowing to take cold medicine for a cold | E277 Improvement of blood glucose lipids and obesity through exercise |
| E134 Conventional medicine and autoimmunity to fight fevers and colds | E278 Acupuncture and massage can be soothing and affect mood |
| E135 Obtaining health information prevention from textbooks or online | E279 Cupping can remove dampness |
| E136 Health is the lack of effects on normal life | E280 Moderate scientific exercise based on one’s condition |
| E137 Understanding exercise habits and knowing how to relax and rest | E281 Pursuing a healthy diet differs between individuals |
| E138 Different populations have different health needs | E282 Exercise improves mental and physical stamina |
| E139 Not shying away from contact with others, being confident and generous | E283 Healthy lifestyles include work, rest, diet, and scientific exercise |
| E140 Avoiding high-calorie junk food | E284 Positive attitudes at work reflect mental health |
| E141 Maintaining a diet of three meals a day | E285 Brushing your teeth at night before going to bed and rinsing your mouth in the morning |
| E142 Staying healthy is the foundation for getting in shape | E286 Nutritional balance to maintain a dynamic balance between energy intake and output |
| E143 Health is having the energy to go about your daily life without getting sick | E287 Endocrine disorders caused by staying up late and eating irregularly |
| E144 Schools offer health programs to give students access to health information | E288 Invisible sub-health states are unhealthy |

**Table S2.** Conceptualization results of 288 tagged labels

| **Number** | **Tagged Labels** | **Number of Materials** | **Number of**  **Participants** |
| --- | --- | --- | --- |
| D1 | 4 articles: E78, E131, E187, E244 | 8 | 4 |
| D2 | 2 articles: E2, E186 | 9 | 5 |
| D3 | 4 articles: E67, E88, E98, E168 | 18 | 10 |
| D4 | 8 articles: E25, E70, E72, E81, E82, E153, E167, E169 | 21 | 10 |
| D5 | 5 articles: E16, E23, E24, E42, E127 | 22 | 12 |
| D6 | 4 articles: E1, E89, E90, E145 | 16 | 8 |
| D7 | 4 articles: E46, E224, E234, E245 | 5 | 4 |
| D8 | 6 articles: E40, E108, E135, E204, E211, E236 | 18 | 12 |
| D9 | 7 articles: E77, E175, E197, E203, E205, E235, E274 | 12 | 6 |
| D10 | 10 articles: E37, E128, E154, E172, E188, E208, E223, E225, E233, E272 | 18 | 10 |
| D11 | 3 articles: E15, E20, E21 | 7 | 3 |
| D12 | 2 articles: E263, E267 | 5 | 1 |
| D13 | 2 articles: E18, E177 | 5 | 4 |
| D14 | 3 articles: E32, E100, E260 | 6 | 4 |
| D15 | 2 articles: E95, E271 | 5 | 4 |
| D16 | 2 articles: E192, E215 | 9 | 4 |
| D17 | 14 articles: E7, E8, E68, E76, E106, E122, E146, E149, E171, E189, E237, E239, E253, E280 | 24 | 12 |
| D18 | 11 articles: E6, E79, E80, E83, E94, E107, E114, E119, E133, E134, E257 | 20 | 10 |
| D19 | 3 articles: E43, E159, E191 | 4 | 3 |
| D20 | 2 articles: E73, E238 | 5 | 5 |
| D21 | 5 articles: E28, E147, E214, E256, E287 | 16 | 11 |
| D22 | 15 articles: E14, E26, E33, E34, E35, E36, E38, E91, E99, E160, E161, E252, E255, E278, E284 | 15 | 10 |
| D23 | 9 articles: E103, E105, E179, E206, E262, E269, E279, E283, E285 | 12 | 7 |
| D24 | 8 articles: E44, E120, E124, E182, E193, E276, E277, E282 | 15 | 10 |
| D25 | 5 articles: E41, E96, E118, E174, E273 | 9 | 6 |
| D26 | 6 articles: E31, E144, E150, E164, E241, E251 | 13 | 5 |
| D27 | 5 articles: E157, E176, E183, E195, E210 | 6 | 3 |
| D28 | 11 articles: E9, E10, E11, E12, E13, E126, E148, E212, E219, E231, E275 | 20 | 7 |
| D29 | 9 articles: E50, E65, E125, E151, E152, E200, E228, E229, E240 | 15 | 8 |
| D30 | 4 articles: E3, E29, E158, E199 | 6 | 3 |
| D31 | 5 articles: E75, E232, E243, E266, E268 | 12 | 9 |
| D32 | 2 articles: E87, E93 | 2 | 2 |
| D33 | 2 articles: E53, E129 | 5 | 3 |
| D34 | 15 articles: E102, E104, E136, E143, E181, E190, E246, E248, E250, E254, E258, E261, E265, E270, E288 | 16 | 10 |
| D35 | 2 articles: E39, E220 | 3 | 3 |
| D36 | 1 article: E242 | 1 | 1 |
| D37 | 3 articles: E137, E221, E259 | 5 | 3 |
| D38 | 4 articles: E54, E84, E85, E196 | 6 | 5 |
| D39 | 2 articles: E222, E247 | 4 | 3 |
| D40 | 2 articles: E166, E170 | 2 | 1 |
| D41 | 1 article: E47 | 1 | 1 |
| D42 | 3 articles: E22, E30, E130 | 6 | 4 |
| D43 | 3 articles: E71, E202, E216 | 3 | 3 |
| D44 | 3 articles: E19, E110, E227 | 8 | 5 |
| D45 | 3 articles: E48, E69, E218 | 9 | 6 |
| D46 | 8 articles: E51, E58, E59, E112, E115, E121, E194, E201 | 17 | 9 |
| D47 | 3 articles: E5, E17, E178 | 6 | 3 |
| D48 | 4 articles: E4, E184, E209, E249 | 6 | 5 |
| D49 | 1 article: E45 | 2 | 1 |
| D50 | 3 articles: E56, E63, E64 | 4 | 3 |
| D51 | 2 articles: E116, E139 | 4 | 2 |
| D52 | 9 articles: E92, E111, E140, E141, E156, E165, E185, E264, E281 | 16 | 9 |
| D53 | 3 articles: E27, E109, E286 | 8 | 5 |
| D54 | 5 articles: E66, E74, E180, E198, E226 | 15 | 9 |
| D55 | 10 articles: E49, E55, E57, E61, E62, E97, E155, E162, E207, E213 | 19 | 12 |
| D56 | 1 article: E52 | 7 | 4 |
| D57 | 3 articles: E132, E163, E173 | 8 | 4 |
| D58 | 5 articles: E60, E117, E138, E142, E230 | 15 | 9 |
| D59 | 2 articles: E113, E217 | 16 | 11 |
| D60 | 3 articles: E86, E101, E123 | 3 | 3 |
| 288 articles | | 593 | 349 |

**Table S3.** Results of concept categorization

| **Subsidiary Category** | **Concept** |
| --- | --- |
| C1  Obtaining Health Information (7) | D3 Can obtain health information from school  D5 Can obtain health information from people around them  D8 Can obtain health-related information on the Internet  D10 Can obtain health information through social practice experience  D16 Can strengthen the mastery of health information and improve health literacy through examinations  D35 Can obtain health information from WeChat public platforms  D44 Focus on health information related to oneself |
| C2  Comprehending Health Information (7) | D7 Can understand health information in the context of their own experiences  D9 Can identify true and valid health information  D11 Can consult a teacher or professional to help understand health information  D34 Can interpret health information correctly  D37 Can perform standardized exercise movements that promote good health  D59 Exercise experiences can help understand related chronic sports injuries and diseases  D60 Health is relevant to sport and is easily understood |
| C3  Perceiving Health Functions (7) | D4 Physiological physical health is a basic state of life  D22 Mental health problems affect physical health status  D32 Addressing health problems requires knowledge of health  D49 Visual external devices can be used to monitor one’s health status  D51 Ability to socialize and have good interpersonal relationships is a sign of mental health  D55 Regular physical activity can regulate the state of mental health  D56 Good health can relieve the mental stress of training or studying. |
| C4  Shaping Health Awareness (9) | D12 Health publicity can draw the attention of college students to their health  D15 Warnings can draw the attention of college students to health awareness  D18 Improving one’s knowledge of prevention of routine diseases and medication indicates health awareness  D19 Taking courses can increase health awareness  D26 Schools can provide health intervention activities to increase health awareness  D39 Declining health among college students highlights the importance of health  D41 Health status varies based on physical quality  D46 Physical exercise can enhance physical fitness and related motor skills  D48 Long-term healthy lifestyles and behaviors can increase health awareness |
| C5  Participating in Health Communication (7) | D1 Can utilize new media communication tools  D2 Health messages communicated by individuals in good health are persuasive  D13 Good communication skills are required to persuade others when communicating health information  D14 Ability to ensure the accuracy of the health information they disseminate  D27 Directly disseminating health information in school-organized health promotion activities  D36 The means and channels of communication are required to share health information  D42 Take the initiative to share and exchange health information with others when encountering health problems |
| C6  Forming Health Motivation (5) | D24 Participate in sports regularly to maintain health  D25 Maintain one’s health to satisfy self-esteem and others’ opinion  D28 Exercise to relieve and improve physical ailments  D40 Varied health motivations lead to different lifestyles  D58 Various groups of people participate in sports for high pursuits, such as getting into shape |
| C7  Making Healthy Decisions (9) | D6 Professional knowledge of healthy exercise is required to conduct scientific exercises  D17 Ability to develop exercise habits in daily life according to one’s situation  D20 Having good social interaction skills can help make correct health decisions  D21 Eating breakfast and not snacking is a healthy dietary habit  D33 Lifestyle habits related to healthcare can affect physical health  D38 Physical exercise can improve physical shape  D45 Exercise experience can help identify health problems, such as sports injuries  D47 Good preparation before exercise can prevent sports injuries |
| C8  Practicing Healthy Living (9) | D23 Healthy living requires the adoption of behavioral habits such as diet, rest, and exercise  D29 Long-term exercise habits can improve physical fitness and prevent chronic diseases  D30 Healthy living habits and exercise behaviors require strong self-discipline  D31 Pre-exercise warm-up and post-exercise stretching are effective in preventing sports injuries  D43 Ability to acquire knowledge of health-promoting exercises  D50 Regular participation in physical activities can improve social interaction skills  D52 Health management requires focus on diet and food combinations  D53 Dynamic balance of energy intake and output can promote health  D54 Maintaining a regular routine and not staying up late |
